# Cellular Stress: Modulator of Regulated Cell Death

**DOI:** 10.3390/biology12091172

**Published:** 2023-08-25

**Authors:** Prem Prasad Lamichhane, Parimal Samir

**Affiliations:** Department of Microbiology and Immunology, University of Texas Medical Branch, Galveston, TX 77555, USA

**Keywords:** apoptosis, GCN2, HRI, integrated stress response, necroptosis, programmed cell death, PERK, PKR, pyroptosis, stress granules

## Abstract

**Simple Summary:**

Stress and regulated cell death signaling are activated simultaneously during disease conditions. However, the crosstalk between the two signaling pathways is poorly understood. There has been a renewed focus on understanding this crosstalk. This review summarizes the current state of knowledge about the crosstalk between stress and regulated cell death.

**Abstract:**

Cellular stress response activates a complex program of an adaptive response called integrated stress response (ISR) that can allow a cell to survive in the presence of stressors. ISR reprograms gene expression to increase the transcription and translation of stress response genes while repressing the translation of most proteins to reduce the metabolic burden. In some cases, ISR activation can lead to the assembly of a cytoplasmic membraneless compartment called stress granules (SGs). ISR and SGs can inhibit apoptosis, pyroptosis, and necroptosis, suggesting that they guard against uncontrolled regulated cell death (RCD) to promote organismal homeostasis. However, ISR and SGs also allow cancer cells to survive in stressful environments, including hypoxia and during chemotherapy. Therefore, there is a great need to understand the molecular mechanism of the crosstalk between ISR and RCD. This is an active area of research and is expected to be relevant to a range of human diseases. In this review, we provided an overview of the interplay between different cellular stress responses and RCD pathways and their modulation in health and disease.

## 1. Introduction

On the first of July 1858, at the meeting of the Linnean Society in London, Charles Darwin’s theory of evolution by natural selection was presented along with Alfred Russel Wallace’s manuscript entitled “Evolution through natural selection of the fittest individuals” [1]. Organisms come across changing external or internal environments, and only the fittest survive. The original concept of the survival of the fittest can be adopted at a cellular level. Cells have sophisticasted physiological mechanisms to sense environmental stress and initiate an adaptive mode; if successful, they survive, otherwise, death is inevitable.

Cells are exposed to various external stressors such as temperature fluctuations, infections, nutrient deprivations, radiation, chemicals, and toxins. In addition, changes in redox states, ionic imbalances, accumulation of unfolded proteins, oncogene activation, etc., contribute to intrinsic cellular stress [2,3,4,5,6,7]. Various signaling pathways that are activated during stress, such as the heat shock response (HSR), unfolded protein response (UPR), DNA damage response (DDR), and response to oxidative stress, efficiently revert the stress-induced alterations to restore cellular homeostasis and prevent cell death.

Cell fate decisions under stress are context dependent and depend on the nature and duration of the trigger [8]. Cells survive when pro-survival pathways are turned ON. Cells entering a pro-death mode activate one of the regulated cell death (RCD) pathways. We focus on apoptosis, necroptosis, and pyroptosis because they have been studied in greater detail than other RCD pathways [9,10,11]. In the current review, we are highlighting cellular stress response mediated translation arrest by various stress kinases in tumor microenvironments (TME) and viral infections. We also discuss how the stress response is being modulated in tumors and infected cells. Finally, we highlight the role of the cellular stress response, especially stress granules (SG), in modulating cell survival, signaling, and RCD.

## 2. Cellular Stress Response and Translation

Cellular homeostasis is maintained by a balance between cell survival and cell death. The rate of physiological cell turnover, in which replicative aged terminally differentiated cells are being continuously replaced (except neurons), varies with cell types [6,12,13,14]. Cells experience replicative stress during the regenerative process, eventually leading to cellular senescence and death of dysfunctional cells [15,16]. In contrast, cells exposed to a wide range of exogenous and endogenous stressors respond to resolve them before initiating a thermodynamically costly RCD pathway. Eukaryotes have a common adaptive signaling pathway named the integrated stress response (ISR) to deal with cellular stress. The ISR pathway reprograms cellular translational machinery to promote the survival of stressed cells [17,18,19].

The complex pathway of translation consisting of four steps, namely, initiation, elongation, termination and ribosome recycling, has been extensively reviewed by others [20,21]. Initiation of translation involves the formation of the 43S pre-initiation complex (PIC) in parallel to the formation of the eukaryotic initiation factor 4F (eIF4F) and the cap binding complex (eIF4F-CBC). Regulation of protein synthesis at the translation initiation step is a major component of translational control. Owing to the fact that translation initiation involves the formation of two independent complexes (PIC and eIF4F-CBC), its regulation can be eIF2α dependent or eIF2α independent [22]. Both of these complexes can be targeted by ISR to repress translation.

**Figure 1:**
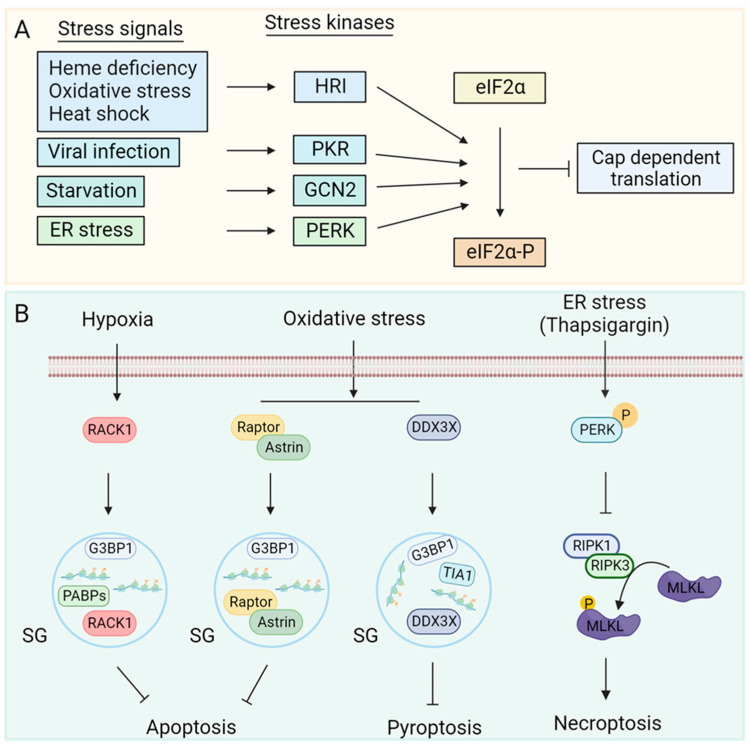
Regulation of stress kinases: (**A**) Heme deficiency, oxidative stress, heat shock, starvation, viral infection, and ER stressors activate HRI, GCN2, PKR and PERK kinases, respectively. Kinase activation phosphorylates eIF2α leading to attenuation of cap dependent translation [23,24,25,26]. (**B**) Cellular stress leads to an increase in local concentration of mRNAs and ribonucleoproteins. RNA binding proteins such as T-cell intracellular antigen 1 (TIA1), GTPase-activating protein-(SH3 domain)-binding protein1 (G3BP1) and poly(A) binding proteins (PABPs) act as nucleation factors for stress granule (SG) assembly [36,37,38]. SGs can sequester proteins involved in apoptosis [4,10] and pyroptosis [9] to inhibit them. ISR can inhibit necroptosis by reducing RIPK3 mediated phosphorylation of MLKL through an unknown mechanism. ER stress activates stress kinase PERK [11] and inhibits phosphorylation of RIPK1 at S166, which inhibits downstream signal transduction to RIPK3 and MLKL, restricting necroptosis [11].

Diverse stress insults activate the distinct ISR kinases [23,24,25,26], namely, General control nonderepressible 2 (GCN2), double-stranded RNA dependent serine/threonine protein kinase R (PKR), PKR-like endoplasmic reticulum kinase (PERK) and heme regulated inhibitor (HRI) kinase that phosphorylates eIF2α leading to translation arrest (Figure 1A).

Amino acid deprivations or defects in tRNA synthetase activity increases cellular levels of uncharged tRNAs. Recognition of uncharged tRNAs by the tRNA synthetase domain of GCN2 activates its kinase activity [27]. Double-stranded RNA generated during viral replication activates PKR [24,28]. Physiological or pathological conditions that increase the accumulation of mistranslated or unfolded proteins lead to ER stress-mediated activation of PERK [25,29]. Heme deficiency [26,30], oxidative stress [26,31] and heat shock [32,33,34] activate the HRI kinase. The ISR kinases are regulated by distinct stressors; however, their catalytic kinase domain phosphorylates eIF2α, allowing stress signals to converge on translation initiation inhibition, which is a critical step in translational control. The affinity of eIF2B for phosphorylated eIF2α-GDP is 150-fold higher than that for eIF2α-GDP [35], which allows inhibition of translation initiation.

Stress-mediated translation arrest increases the local concentration of mRNA and 40S ribosome-containing ribonucleoproteins (mRNPs) and subsequent sequestering of mRNPs in membraneless cytoplasmic foci called SGs. RNA binding proteins (RBPs) such as T-cell intracellular antigen-1 (TIA1), TIA1-related protein (TIAL1), poly(A) binding proteins (PABPs), GTPase-activating protein-(SH3 domain)-binding protein 1 (G3BP1) and several DEAD-box proteins promote SG assembly [36,37,38,39]. In addition to mRNPs, SGs also sequester central components of the RCD pathways that modulate apoptosis [4,10], pyroptosis [9], and necroptosis [40] mediated cell death (Figure 1B).

## 3. Regulated Cell Death

**Figure 2:**
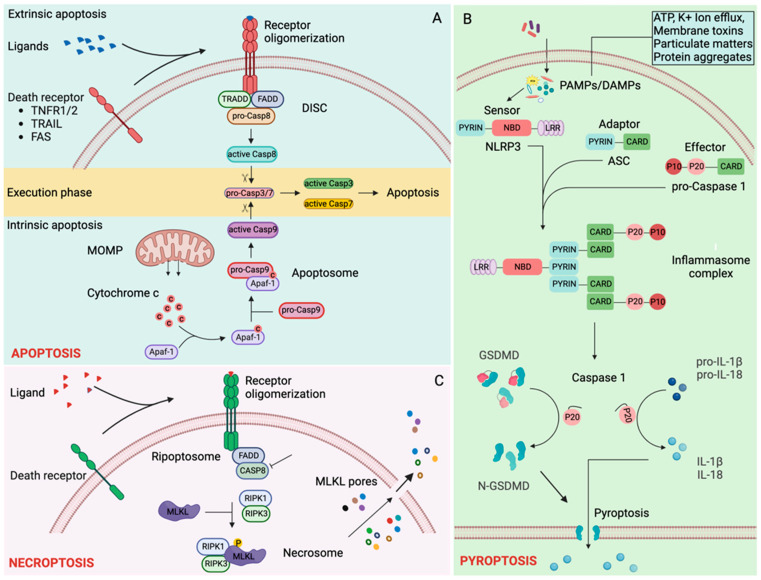
Regulated cell death pathways: (**A**) Apoptosis. Ligand binding induces oligomerization of death receptors activating the extrinsic apoptosis cascade. Tumor necrosis factor receptor 1 (TNFR1), TNFR1/2-associated death domain protein (TRADD), Fas-associated death domain protein (FADD) and pro-caspase-8 are recruited to the oligomerized death receptors forming death-inducing signaling cascade (DISC). Autoproteolytic processing of pro-caspase 8 generates catalytically active initiator caspase 8, which cleaves executioner caspases, caspase-3 and caspase-7, completing extrinsic apoptosis [41,42,43]. Intrinsic apoptosis is triggered by various intracellular danger signals that induce an increase in mitochondrial outer membrane permeabilization (MOMPs). An increase in MOMPs releases cytochrome c in the cytosol. Cytochrome c binds with APAF1 and pro-caspase-9 forming an apoptosome signaling complex. Autoproteolytic processing of pro-caspase-9 in the apoptosome generates catalytically active initiator caspase-9, which cleaves executioner caspases, caspase-3 and caspase-7, leading to intrinsic apoptosis [41,42,43]. (**B**) Pyroptosis. Diverse PAMPs and DAMPs are sensed by different inflammasome sensors, NLRP1 [44], NLRP3 [45,46,47,48,49,50,51], NLRC4 [52,53,54], PYRIN [55,56], and AIM2 [57,58,59,60]. Activated inflammasome sensors mediate ASC (apoptosis-associated speck-like protein containing CARD) dependent recruitment of pro-Caspase 1 and subsequent autoprocessing generating active caspase-1 [61,62,63,64]. Active caspase-1 cleaves pro-interleukin-1β (proIL-1β) [65,66,67], pro-IL-18 [68,69] and Gasdermin D (GSDMD) [70,71]. The N-terminal fragment of GSDMD forms pyroptotic pores through which IL-1β and IL-18, along with intracellular content, are released [70,72]. (**C**) Necroptosis. TNFR1 signaling in the absence of caspase-8 recruits RIPK1 (receptor-interacting protein kinase 1) and RIPK3 forming a necroptosis-inducing complex called the necrosome [42,73]. MLKL (mixed lineage kinase domain-like pseudokinase) is phosphorylated at the necrosome and translocates to the cell membrane to form pores that cause osmotic lysis of cells [74,75,76].

Cell death is a normal physiological process. However, during infections and several pathological conditions, pathogen-associated molecular patterns (PAMPs) or damage-associated molecular patterns (DAMPs) elicit robust immune responses, which can be detrimental to the host. Therefore, cells have developed a regulated death system to modulate the immune response. Thus, cell death can be immunologically silent, such as apoptosis or immunogenic, and pyroptosis or necroptosis (Figure 2). Several RCD pathways have been described that differ in their mechanisms of activation and effect on host homeostasis. In this review, we will focus on apoptosis, necroptosis, pyroptosis, and ferroptosis because their regulation by ISR and SGs has been studied the most.

### 3.1. Apoptosis

Apoptosis plays a crucial role in physiological cell turnover. Various pathological conditions or insults may lead to apoptotic cell death. The trigger for apoptosis can be extrinsic or intrinsic (Figure 2A).

Binding of death ligands such as tumor necrosis factor (TNF), fas ligand (FASL), or TNF-related apoptosis-inducing ligand (TRAIL) to their cognate cell surface death receptors, TNF receptor 1 (TNFR1), FAS, or TRAIL receptor 1/2 (TRAILR1/2), respectively, leads to receptor oligomerization, which initiates recruitment of adaptor proteins TRADD (TNFR1/2-associated death domain protein), and FADD (Fas-associated death domain protein). This is followed by FADD-mediated recruitment of the initiator caspase-8 (CASP8), forming a signaling platform called DISC (Death-inducing signaling complex). The formation of DISC initiates autocatalytic proteolysis and activation of CASP8, triggering the execution phase of apoptosis [41,42].

Mitochondrial outer membrane permeabilization (MOMP) initiates intrinsic apoptosis by releasing the pro-apoptotic protein cytochrome c from the mitochondrial intermembrane space into the cytoplasm [77]. Cytochrome c binds to APAF1, which recruits the initiator caspase-9 (CASP9) to form a complex called apoptosome. Autocatalytic proteolysis and activation of CASP9 in the apoptosome initiate the execution phase of apoptosis [41,42].

Cleaved initiator caspases (CASP8 and CASP9) proteolytically activate executioner caspases, caspase-3 (CASP3) and caspase-7 (CASP7). Active executioner caspases cleave their target proteins, including cytoskeletal components and are responsible for the morphological and biochemical alterations that ultimately result in apoptosis [43].

### 3.2. Pyroptosis

Pyroptosis is an inflammasome-dependent inflammatory cell death pathway mediated by the pore-forming protein gasdermin d (GSDMD). Inflammasome sensors such as Nucleotide oligomerization domain (NOD) like receptor (NLR) pyrin (PYD) domain-containing protein 1 (NLRP1) [44], NLRP3 [45,46,47,48,49,50,51], NLR family caspase activation and recruitment domain (CARD) containing protein 4 (NLRC4) [52,53,54], absent in melanoma 2 (AIM2) [57,58,59,60], and PYRIN [55,56] sense different PAMPs and DAMPs (Figure 2B). Activated inflammasome sensors oligomerize, forming a signaling platform to which apoptosis-associated speck-like protein containing a CARD (ASC, also known as PYCARD) is recruited, which in turn recruits caspase-1 (CASP1) where it gets activated by proximity-induced autoproteolysis [61,62,63,64]. NLRP1 and NLRC4 sensors themselves contain a CARD domain and are capable of recruiting CASP1 independent of ASC [78,79]. Active CASP1 cleaves GSDMD [70,71], interleukin-1β (IL-1β) [65,66,67] and IL-18 [68,69] among others. The N-terminal fragment of GSDMD (GSDMD-N) translocates to the plasma membrane to form pores that result in the osmotic lysis of cells. GSDMD-N pores also facilitate the release of several cytosolic inflammatory mediators, including active IL-1β and IL-18, in the absence of cell lysis [70,72].

### 3.3. Necroptosis

Necroptosis is a regulated form of cell death originally found to be triggered by the activation of TNFR, leading to mixed lineage kinase domain-like pseudokinase (MLKL) activation downstream of RIPK3 (receptor-interacting protein kinase 3) (Figure 2C). One of the most studied necroptosis occurs in cells when CASP8 is inhibited in the presence of TNFR1 activation [42,73]. TNFR1 signaling (in the absence of CASP8) recruits RIPK1, which interacts with RIPK3 through the RHIM (RIP homotypic interaction motif) domain. RIPK1 and RIPK3 form a complex called necrosome. MLKL is recruited and phosphorylated in the necrosome. The phosphorylated MLKL is trafficked to the plasma membrane, where MLKL oligomers execute cell death through the disruption of plasma membranes [11,42,74,75,76]. Depending on the stimuli, other RHIM domains containing proteins such as Z-DNA binding protein 1 (ZBP1) and Toll–IL-1 receptor domain-containing adaptor-inducing IFN-β (TRIF) can interact with RIPK1 [42].

## 4. Stress Kinases in Regulation of Apoptosis

### 4.1. GCN2 in Regulation of Apoptosis

**Figure 3:**
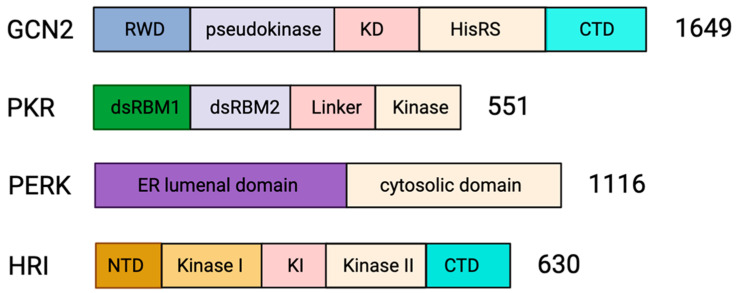
Domain organization of stress kinases. The domain architecture of the four mammalian eIF2α kinases is represented as bars. GCN2 is 1649 amino acids long and has an N-terminal RWD domain (RING finger-containing protein, WD repeat-containing protein and yeast DEAD-like helicase), pseudokinase domain, catalytically active kinase domain (KD), histidyl-tRNA synthetase like (HisRS) domain and C-terminal domain (CTD) [80,81]. PKR is 551 amino acids long and has an N-terminal regulatory domain joined by a linker domain to the catalytic C-terminal kinase domain. The regulatory domain has two double-stranded RNA (dsRNA) binding motifs; dRBM1 and dRBM2 [28,85]. PERK is 1116 amino acids long and consists of an N-terminal regulatory lumenal domain and a C-terminal cytosolic kinase domain [86]. HRI is 630 amino acids in length and has an N-terminal domain, a central regulatory kinase insertion (KI) domain flanked by two kinase domains (Kinase I and Kinase II) and the C-terminal domain [86,87].

Human GCN2 is a 1649 amino acid long protein that forms a homodimer (Figure 3) and has five conserved domains, namely, an N-terminal RWD domain (RING finger-containing protein, WD repeat-containing protein and yeast DEAD-like helicase), a pseudokinase domain, a catalytically active kinase domain (KD), a histidyl-tRNA synthetase like (HisRS) domain and a C-terminal domain (CTD) [80,81]. At a basal metabolic state, the interaction between CTD and KD auto-inhibits the kinase activity of GCN2. Additionally, CTD-CTD and CTD-HisRS interactions stabilize the inactive dimeric architecture of the GCN2 [80,81]. Starvation, in particular amino acid deprivation, increases the amount of deacylated tRNA in cells. In addition, glucose deprivation shunts the amino acids to the cytosolic vacuoles [82,83], increasing the uncharged tRNA pool. The binding of uncharged tRNA to the HisRS domain of GCN2 [80,81,84] weakens the autoinhibitory interactions followed by structural rearrangement that activates the kinase activity of GCN2 [84], leading to eIF2α phosphorylation and translation inhibition.

**Figure 4:**
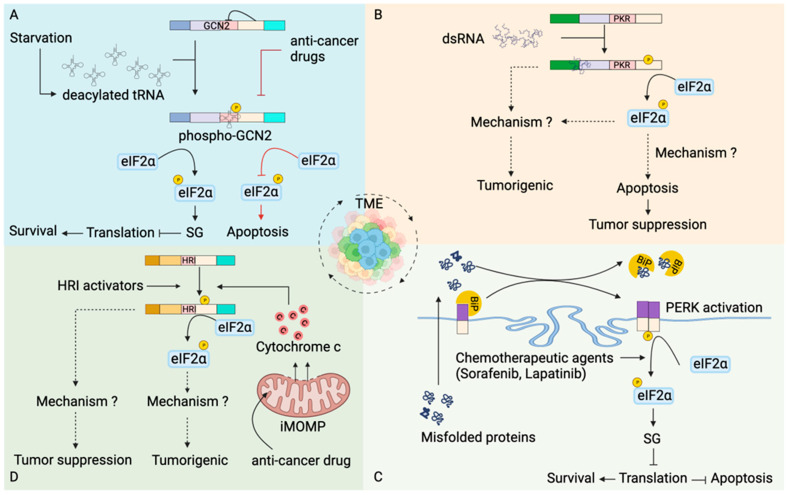
Stress kinases in modulation of apoptosis in tumors. (**A**) Tumor cells have increased expression of GCN2 [90,91,92,93,94,95]. Nutrient deprivation in tumor microenvironment (TME) increases the pool of uncharged tRNAs, which binds to HisRS domain of GCN2. Activated GCN2 phosphorylates eIF2α leading to translational inhibition and promoting survival of tumor cells. In contrast, inhibition of GCN2 by anti-tumor drugs promoted tumor cell death [95,96]. (**B**) dsRNA generated from diverse cellular processes within the TME activates PKR. PKR activation can have tumorigenic [28,97,98,99,100,101,102] as well as tumor-suppressive roles [103,104,105,106]. How PKR promotes tumorigenesis is unknown. PKR-dependent eIF2α phosphorylation mediates apoptosis of the tumor cells [105,106,107] and promotes tumor suppression. (**C**) Stress in TME leads to accumulation of unfolded or misfolded proteins [108], which translocate to the endoplasmic reticulum (ER). An increase in misfolded proteins in the ER leads to dissociation of immunoglobulin binding protein (BiP) from the PERK and subsequently activates PERK [109]. Activated PERK phosphorylates eIF2α leading to formation of SGs. In addition, chemotherapeutic agents also favor eIF2α phosphorylation mediated formation of SGs [110,111,112,113]. SGs formation stalls translation at the initiation step, inhibits apoptosis and favors tumor cell survival. (**D**) Compounds such as *N*,*N*′-diarylureas activate HRI leading to eIF2α phosphorylation-dependent inhibition of oncogenesis [114,115,116]. Chemotherapeutic agents induce incomplete mitochondrial outer membrane permeabilization (iMOMPs), liberating suboptimal cytochrome c, which activates HRI kinase [117]. HRI kinase-mediated translational reprogramming generates drug-resistant cancer cells that promote tumorigenesis.

Cellular metabolism in normal cells maintains physiological homeostasis during cell growth and proliferation. In contrast, uncontrolled cell proliferation during malignancies leads to nutrient deficiencies and metabolic stress. However, metabolic reprogramming occurs in cancer cells [88,89] to provide nutrients for the growth of tumor cells (Figure 4A). As such, GCN2 is highly expressed in papillary renal cell carcinoma [90], head and neck small cell carcinoma [91], prostate cancer [92], cisplatin-resistant human gastric cancer cells [93], hematological malignancies such as multiple myeloma, acute myeloid leukemia, and acute lymphoid leukemia [94,95], hepatocellular carcinoma [96] and pancreatic cancer cells [95]. Inhibition of GCN2 leads to an increase in apoptosis of tumor cells [95,96], suggesting that the stress kinase GCN2 helps malignant cells to cope with nutrient limitation favoring tumor growth.

**Figure 5:**
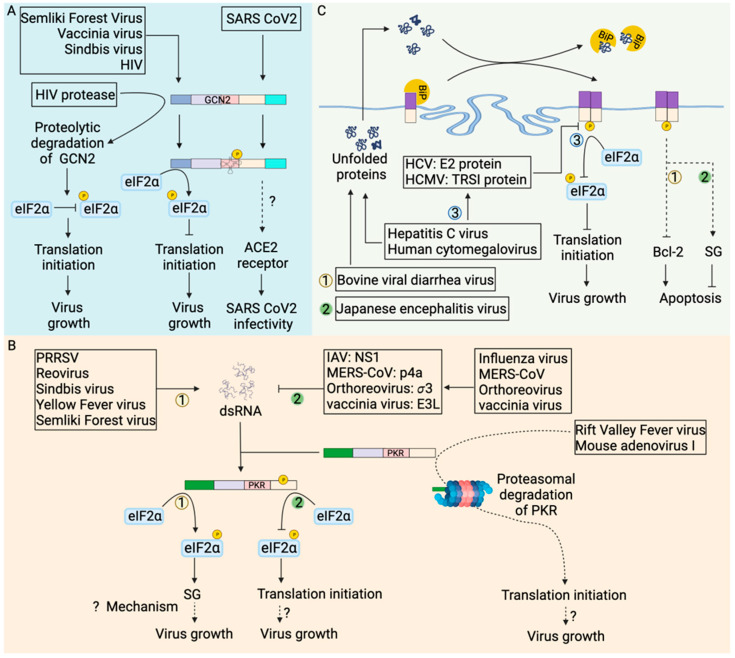
Stress kinases in modulation of viral infection outcome. (**A**) Virus infection activates GCN2 leading to eIF2α phosphorylation mediated translation shutdown, which inhibits virus replication [121,122]. In contrast, HIV-1 proteases cleave GCN2, abrogating eIF2α phosphorylation mediated translation inhibition and promoting virus growth [122]. Moreover, GCN2 phosphorylation during SARS coronavirus 2 infection increases expression of the ACE2 (Angiotensin-converting enzyme 2) receptor [120] that increases SARS-CoV2 infectivity. (**B**) dsRNA generated during virus replication activates PKR. PKR-mediated eIF2α phosphorylation inhibits translation leading to the formation of stress granules favoring virus replication through poorly explored mechanisms. Rift valley fever virus [123,124] and mouse adenovirus type I [125] routes activated PKR for proteasomal degradation to favor its replication. Viral proteins such as influenza virus nonstructural protein 1 (NS1) [126], MERS-CoV p4a protein [127], mammalian orthoreovirus *σ*3 protein [128], and vaccinia virus E3L protein [129] sequestered viral dsRNA away from PKR abrogating PKR activation to favor virus replication. (**C**) Profound virus replication generates unfolded or misfolded proteins which translocate to the endoplasmic reticulum (ER). An increase in misfolded proteins in the ER leads to dissociation of immunoglobulin binding protein (BiP) from the PERK [109]. Hepatitis C virus E2 protein [130] and human cytomegalovirus TRSI protein [131] inhibit the kinase activity of PERK, abrogating eIF2α phosphorylation and favoring virus replication. The bovine viral diarrhea virus mediates PERK-dependent inhibition of anti-apoptotic protein Bcl-2, promoting apoptosis of infected cells [132]. In contrast, Japanese encephalitis virus induces stress granules in a PERK-dependent manner to inhibit apoptosis and promote virus replication [133]. In (**B**,**C**), each number represents the unique pathway being followed (example: number 1 is one pathway while number 2 represents the other pathway).

Viruses also manipulate the host-cell metabolic pathway [118] to provide biomolecules, in particular amino acids, for the production of new virions. However, the role of GCN2 stress kinase (Figure 5A) in regulating viral infection dynamics is contextual. Angiotensin-converting enzyme 2 (ACE2), the receptor for severe acute respiratory coronavirus 2 (SARS-CoV-2), is also involved in the intestinal uptake of amino acids [119]. GCN2 increased expression of SARS-CoV-2 receptor in amino acid starved human colonic epithelial cells [120] aggravates the disease. Semliki Forest virus and vaccinia virus infections increase the kinase activity of GCN2 and subsequent attenuation of viral replication [121]. Genomic RNA of Sindbis virus (SV) [121] or human immunodeficiency virus-1 (HIV-1) [122] activate GCN2 kinase activity which blocks translation, including viral protein synthesis hindering virus replication. It is unclear if SG assembly plays a role in viral replication inhibition in these cases.

Although activation of GCN2 kinase activity and subsequent phosphorylation of eIF2α can lead to SG assembly, the formation of SGs in tumor cells has been challenging to detect. One possible reason might be that the downregulation of SG components in these cells inhibits SG assembly. Another reason could be that the dynamic nature of SG assembly:disassembly processes make it hard to capture them in vivo. The formation of SGs in tumor cells treated with chemotherapeutic agents [110,134] has provided indirect evidence for SG formation. Additionally, viral proteases, such as HIV-1 protease, cleave GCN2 and abrogate translation inhibition [122] to increase viral protein synthesis and viral replication. Taken together, modulation of SG formation might be an alternative approach to alter cancer progression and virus infection outcomes.

### 4.2. PKR in Regulation of Apoptosis

Protein Kinase R (PKR) is a 551 amino acid long monomeric protein consisting of a regulatory N-terminal double-stranded RNA (dsRNA) binding domain and catalytic C-terminal kinase domain joined by a 20 amino acid linker region (Figure 3). The two tandem copies of dsRNA binding motifs, dsRBM1 and dsRBM2, in the N-terminal domain, bind dsRNA in a sequence-independent manner. The binding of dsRNA to the regulatory domain of PKR induces dimerization of the kinase domain and phosphorylation-mediated activation of PKR [28,85]. Activated PKR phosphorylates eIF2α to inhibit translation initiation and induce SG assembly.

PKR has been implicated in the regulation of various cellular processes in tumors (Figure 4B). dsRNA from diverse cellular sources, including those liberated from dying cells in the TME, are subsequently internalized by the tumor cells and are recognized by RNA sensors [135,136], including PKR. Studies have shown a dual role of PKR in tumors, tumor-suppressive or tumorigenic. Initial studies suggested an anti-proliferative role of PKR pertaining to the inhibition of translation initiation [137]. NIH 3T3 cells expressing PKR exhibited a delay in malignant transformation in nude mice [103]. Similarly, PKR downregulated HCT116 human colon cancer cells grew rapidly in nude mice [104], suggesting a tumor-suppressive role of PKR. PKR overexpressing cells undergo Fas-associated death domain (FADD) mediated activation of caspase 8 [105,106,107], leading to apoptosis. Additionally, 3T3 L1 cells expressing catalytically inactive PKR were resistant to apoptosis and underwent malignant transformation [105].

In contrast to the tumor-suppressing role of PKR discussed above, transcriptomics analysis showed overexpression of PKR in several cancer types [28]. Overexpression of PKR or increased PKR activity is seen in pancreatic cancer [28], melanoma [97], colon cancer [97], breast cancer [98] and hepatocellular carcinoma [99], suggesting its role in tumorigenesis. Furthermore, it has been suggested that increased PKR activity promotes tumor progression and invasion [97,100,101,102]. Interestingly, tumors consist of a heterogeneous population of cancer cells [138], and there is spatial heterogeneity of PKR expression, at least in head and neck squamous cell carcinoma [139]. Intriguingly, cancer cells with high PKR expression were present in the core of tumors, while those with low levels were at the periphery [139]. The significance of this observation remains to be unraveled.

The contradicting role of PKR in apoptosis could be due to the differential role of PKR in normal and cancer cells. Although the knockdown of PKR leads to apoptosis of both normal and cancer cells, there was a greater increase in tumor cell apoptosis, suggesting a strong anti-cancer therapeutic potential of PKR inhibition and emphasizing the role of PKR in tumor cell survival [140]. In addition, PKR has been shown to finely tune the balance between the pro-survival and pro-death signal by activating both nuclear factor kappa-B (NF-κB) and eIF2α in a sequential manner [141]. In NIH3T3 cells expressing PKR (tet-off system), NF-κB is activated at the earliest time point, promoting cell survival independent of PKR kinase activity. On the other hand, catalytically active PKR phosphorylates eIF2α at a later time point and subsequently promotes apoptosis [141]. Although sustained elevation of NF-κB activity, a key player in tumorigenesis [142], might explain the survival of cancer cells. The mystery that remains unresolved is “how kinase activity of PKR is being differentially modulated in the tumor cells?”.

PKR is activated by dsRNA, a byproduct of RNA virus replication and some DNA viruses (Figure 5B). Activated PKR phosphorylates eIF2α to attenuate translation leading to formation of stress granules and subsequently triggering apoptosis. However, many viruses avoid the formation of stress granules in host cells confirming that viruses can manipulate PKR-mediated cellular stress responses for their benefit. The Middle East Respiratory Syndrome Coronavirus (MERS-CoV) accessory protein p4a [127], mammalian orthoreovirus *σ*3 protein [128], vaccinia virus E3L protein [129] and influenza A virus nonstructural protein 1 (NS1) [126] bind and sequester dsRNA to prevent PKR activation in infected cells. Viral proteins, for instance, influenza NS1 [143] and vaccinia virus E3L [129] protein, can directly bind with PKR to prevent PKR-mediated eIF2α phosphorylation. In addition, viral proteins can degrade PKR [123,124,125] by mechanisms that are incompletely understood for efficient viral replication. During rift valley fever virus [123,124] and mouse adenovirus type 1 infection [125], PKR is degraded in the proteasome. Overall, viruses evolved mechanisms to inhibit PKR activation avoiding SG formation for efficient virus replication.

Nevertheless, many viruses activate PKR and form SGs. Reovirus [128,144], sindbis virus [145], semliki forest virus [146], yellow fever virus [147] and porcine reproductive and respiratory syndrome virus (PRRSV) [148] infections lead to PKR-mediated formation of SGs in infected cells. PKR-mediated activation of cellular stress response facilitated reovirus replication by relocating viral transcripts to unknown sites where limited cellular translational machinery is accumulated [144]. In contrast, PPRSV replicase protein nsp1β localizes and interacts with PKR within SG in infected cells [148], utilizing SGs for viral benefit. The status of cell death in virus-infected cells that form SGs has not been explored except for Sindbis virus-infected cells where PKR activation leads to inhibition of translation of anti-apoptotic B-cell lymphoma 2 (bcl-2) protein and apoptosis of the infected cells [145].

### 4.3. PERK in Regulation of Apoptosis

PKR-like endoplasmic reticulum kinase (PERK) is an 1116 amino acid long transmembrane protein consisting of an N-terminal endoplasmic reticulum (ER) lumenal domain and a C-terminal cytosolic domain (Figure 3). The regulatory lumenal domain helps in dimerization, while the cytosolic domain has kinase activity and consists of autophosphorylation sites [86]. Immunoglobulin binding protein (BiP) maintains PERK as inactive monomers [109]. However, ER stress causes BiP to dissociate from PERK, facilitating its dimerization and activation of its kinase domain [109]. Activated PERK phosphorylates eIF2α leading to translational arrest [149]. Perturbation of ER homeostasis has been identified in several pathological conditions, including cancer [150], viral infections [151] or neurodegeneration [152].

Cancer cells experience adverse physiological conditions such as hypoxia and nutrient deprivation in the TME. The cumulative effect is the accumulation of unfolded or misfolded proteins in the ER of tumor cells leading to ER stress [108]. Cells respond to deregulated ER homeostasis through the UPR signal, PERK being one of the sensors of ER stress (Figure 4C) [153]. PERK plays a vital role in oncogenesis and tumor progression. Differential expression of PERK is found in several tumors and cancers [153]; neuronal cancer, liver cancer, lung cancer, breast cancer, gastric carcinoma and cholangiocarcinoma have high expression, while thyroid cancer, lymphoma, sarcoma and colorectal cancer have low expression [153] suggesting variation in ER stress among different cancer types. How different levels of ER stress fine-tune the progression of various tumor types is an exciting area for future research. However, another possibility for differential expression exists in the spatial heterogeneity in PERK expression arising from heterogeneous tumor cell populations.

Although tumor cells have sustained expression of PERK, no histological evidence of them forming SGs in an eIF2α kinase-dependent manner exists. Human cervical cancer HeLa cells form SG under hypoxia [111]; this might provide intriguing evidence to implicate SG formation in cancer cells within TME. SG formation helps the cancer cells to survive the extreme physiological conditions of TME and avoid RCD [10,154]. In line with this, treatment with various anti-cancer drugs induces PERK-dependent formation of SGs in tumor cells [110,111,112,113]. Downregulation of PERK sensitizes tumor cells to chemotherapy leading to apoptosis [110,112,113]. Taken together, PERK expression in tumor cells supports oncogenesis.

Viruses are obligate intracellular pathogens and depend on host cell machinery for viral protein synthesis and replication. Virus infection involves the synthesis of viral proteins, which are routed to the ER for proper protein folding and post-translational modifications. In addition, accumulation of unfolded or misfolded viral proteins can occur in the ER and induce ER stress (Figure 5C). Under ER stress, BiP is released from PERK, leading to PERK homodimerization and autophosphorylation-mediated activation of PERK. Activation of PERK blocks translation as a measure to cope with stress. Sustained ER stress can lead to RCD.

Activation of ER stress has been detected in a range of viral infections. Flaviviruses and coronaviruses exploit ER as a site of viral protein synthesis, replication and assembly [155]. PERK autophosphorylation was observed in Madin-Darby Bovine Kidney (MDBK) cells infected with the bovine viral diarrhea virus (BVDV) [132]. Sustained ER stress downregulated anti-apoptotic protein Bcl-2 leading to apoptosis of BVDV-infected cells [132]. Japanese encephalitis virus (JEV), another ER tropic virus, involves the budding of progeny virions from the ER membrane of infected BHK-21 cells and initiates ER stress. However, PERK activation was not detected by mobility shift assay [156]. In contrast, PERK activation is observed by immunoblot in both neuronal cells and BHK-21 cells infected with JEV [133], suggesting variation in PERK activation can be cell type dependent or technique dependent In addition, PERK activation promoted SG formation in JEV-infected neuronal cells, which subsequently repress apoptosis [133].

Transmissible gastrointestinal virus (TGEV) infection of the IPEC-J2 jejunum epithelium induced ER stress. Activated PERK suppressed TGEV replication [157] through phospho-eIF2α mediated translation inhibition. Similar to what has been observed with TGEV, viruses such as human cytomegalovirus (HCMV) [158] and hepatitis C virus (HCV) [130] infections also activate PERK. However, HCV E2 protein binds with PERK to inhibit PERK activity and prevent eIF2α phosphorylation [130]. Similarly, HCMV immediate early protein TRS1 prevents eIF2α phosphorylation [131]. HCMV and HCV, thus, maintain protein translation to benefit virus replication. In summary, PERK is a critical regulator of cellular stress response to ER stress and plays a multifaceted role in tumorigenesis and host response to viral infections.

### 4.4. HRI in Regulation of Apoptosis

Heme-regulated inhibitor (HRI) kinase is a 630 amino acid long protein consisting of five domains, an N-terminal domain, a C-terminal domain, and a central kinase insertion (KI) domain flanked by two kinase domain (Kinase I and Kinase II) [86,87] (Figure 3). The heme binding site is located on the N-terminal and the central KI domain of HRI; however, the binding site on the KI domain performs the regulatory role [87]. Heme binding at the N-terminal heme binding site dimerizes the HRI. The HRI dimer is stable and inactive during heme sufficiency. However, heme deficiency HRI is activated by a mechanism of transactivation that subsequently leads to the phosphorylation of eIF2α to inhibit translation [30,159,160].

HRI being a sensor of heme, modulates erythropoiesis. During heme sufficiency, HRI is inhibited, and protein synthesis (the globin chains of hemoglobin) is activated. In contrast, heme deficiency activates HRI-mediated phosphorylation of eIF2α, which inhibits translation, in particular globin synthesis [31]. Strikingly, HRI senses heme availability to balance heme and the globin chain synthesis by increasing apoptosis of late erythroid precursors during iron deficiency anemia [159].

The expression of HRI is not limited to red blood cells and has been found in macrophages and the liver [86,161], suggesting the role of HRI beyond red blood cells (Figure 4D). Compounds such as *N*,*N*′-diarylureas that activate HRI inhibited the proliferation of tumor cells in an eIF2α phosphorylation-dependent manner [114,115,116]. HRI expression has been found to be high in tissue samples from lungs, ovary, breast and gastric cancers compared to normal tissue and is associated with poor survival [117]. Targeting HRI to trigger apoptosis might be a new strategy for anti-cancer therapy. However, following anti-cancer therapy, drug-tolerant persister cells are generated, which are dependent on sublethal cytochrome c (released due to incomplete MOMPs) mediated activation of HRI [117], suggesting translational reprogramming by HRI can promote tumorigenesis.

## 5. Emerging Concept on Crosstalk between Cellular Stress and Other Forms of Cell Death

**Figure 6:**
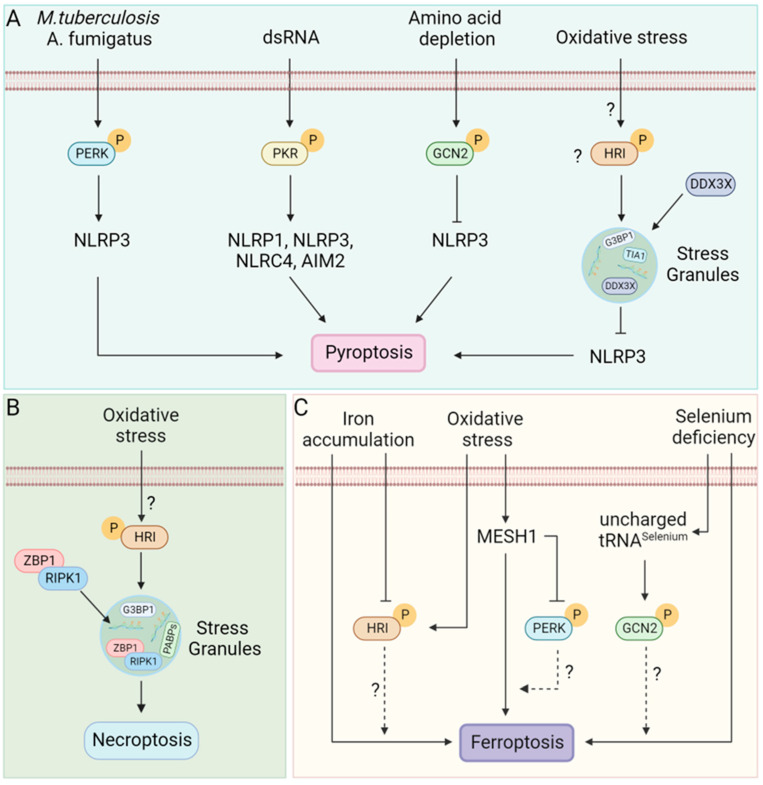
RCD pathways and Stress kinases. (**A**) *M. tuberculosis* [167] and *A. fumigatus* [168] infection induces ER stress-mediated PERK phosphorylation. Double-stranded RNA (dsRNA) phosphorylated PKR [169]. Phosphorylated PERK and PKR can activate inflammasomes leading to pyroptosis [167,168,169]. In contrast, amino acid depletion during inflammation phosphorylates GCN2, which suppresses inflammasome activation, reducing pyroptosis [170]. Additionally, SGs formed in response to several stressors, including arsenite-induced oxidative stress sequester DDX3X and inhibit NLRP3 inflammasome activation [9]; however, whether HRI alone can suppress activation of the NLRP3 inflammasome or other stress kinases are also capable is unclear. (**B**) Oxidative stress phosphorylates HRI leading to translation arrest and formation of SGs. SGs act as a site for necrosome assembly that activates necroptosis [40]. (**C**) Selenium deficiency sensitizes cells to ferroptosis [171,172]; uncharged tRNA during selenium deficiency might activate the amino acid sensor GCN2 that possibly modulates the outcome of ferroptosis. Oxidative stress induces MESH1 (Metazoan SpoT Homologue 1), leading to cell death by ferroptosis [173,174]. In addition, MESH1 can inhibit PERK activation [174]; the crosstalk between PERK activation and ferroptosis is yet to be discovered. Cellular iron accumulation activates ferroptosis [175,176]. How stress kinases that sense oxidative stress or heme interconnect cellular stress with ferroptosis is unknown.

The eIF2α kinases have long been known for their ability to modulate cellular homeostasis during stress, often activating apoptosis if the stress persists beyond a threshold. Apoptosis is not the only RCD program that can be triggered; cells can die by pyroptosis, necroptosis or other more recently discovered RCD pathways. RCD is a very active area of research. This has led to the discovery of several RCD pathways in the last decade [162]. Since then, other RCD pathways have been proposed, including PANoptosis, cuproptosis, oxeiptosis and ferroptosis [163,164,165,166]. The crosstalk between stress and most of these RCD pathways is poorly understood (Figure 6). In this review, we focus on apoptosis, necroptosis, pyroptosis, and ferroptosis and discuss their modulation by ISR since we have a better, albeit still incomplete, understanding of them.

### 5.1. Stress and Pyroptosis

The stress kinase PKR plays a crucial role in the activation of inflammasomes [169,177,178,179,180]. PKR physically interacted with NLRP1, NLRP3, NLRC4 and AIM2 inflammasome and induced activation of caspase 1 and IL-1β cleavage [169]. Inhibition of PKR activity suppressed inflammasome activation and IL-1β release [169,177,178,179,180]. In contrast, another study could not establish the role of PKR in inflammasome activation [181]. However, multiple lines of evidence suggested crosstalk between stress kinase PKR and pyroptosis. The induction of ER stress has been found to activate NLRP3 inflammasome activation and pyroptosis during fungal infection [168] and infection with *Mycobacterium tuberculosis* [167] (Figure 6A).

In contrast, activation of the GCN2/eIF2α pathway has been shown to suppress inflammasome activation, resulting in reduced pyroptosis during intestinal inflammation [170]. Additionally, induction of SGs has been shown to inhibit NLRP3 inflammasome [9] wherein SGs sequestered DDX3X activating a pro-survival mode; in contrast, the association of DDX3X with NLRP3 induces pyroptosis [9,182]. In summary, stress signaling has a pleiotropic effect on pyroptosis (Figure 6A). The molecular mechanisms of this pleiotropic effect are poorly understood and are an active area of research due to their immense therapeutic potential.

### 5.2. Stress and Necroptosis

RNA viruses and some DNA viruses replication generate dsRNA, which can adopt a right-handed conformation (A-RNA) or a left-handed conformation (Z-RNA). PKR and melanoma differentiation-associated protein 5 (MDA5) senses A-RNA [183], while Z-DNA binding protein 1 (ZBP1) senses Z-RNA [184]. Adenosine deaminase acting on RNA 1 (ADAR1) performs adenosine-to-inosine base editing of endogenous dsRNA [185] to prevent PKR [186] or MDA5 overactivation [187] and spontaneous activation of ZBP1 [184]. Accumulation of Z-RNA during infection with viruses such as influenza, SARS-CoV2, murine cytomegalovirus and vaccinia triggers ZBP1-RIPK3-mediated necroptosis [188,189,190,191,192]. In addition, ZBP1 is a key regulator of tumor cell necroptosis [193,194].

ADAR1, as well as ZBP1, the key molecules of the necroptotic pathway, localizes to arsenite-induced SGs (Figure 6B) in HeLa cells and L929 cells, respectively [40,195]. In the SG, ZBP1 forms ZBP1-RIPK3 necrosomes triggering necroptosis [40]. In contrast, various stressors (thapsigargin, brefeldin A, tunicamycin, MG132) inhibited necroptosis (Figure 6B) in bone marrow-derived macrophages [11]. The contrasting result might be due to differences in stressors used and the cell type under study.

Nutrient deprivation (glucose or serum) induces ZBP1-mediated necroptosis of breast cancer cells [194], suggesting ZBP1 expression is increased during starvation. However, tumor cells execute metabolic reprogramming to provide nutrients for proliferating cancer cells and possibly downregulate ZBP1 to prevent necroptosis. In agreement, increased ZBP1 expression correlated with breast cancer aggressiveness [196].

Taken together, these data support the notion that there is a crosstalk between necroptosis and SG. However, our understanding of the mechanism of this crosstalk remains in its infancy. Future research on this topic will greatly aid in the development of therapeutics against a range of infectious and inflammatory diseases.

### 5.3. Stress and Ferroptosis

Ferroptosis is an iron-dependent caspase-independent form of regulated cell death activated by oxidative stress [166]. The peculiar feature of ferroptosis is the peroxidation of membrane phospholipids leading to membrane rupture culminating in cell death [197]. Ferroptosis is inhibited by reduced glutathione (GSH) and *glutathione peroxidase 4* (GPX4) [198,199]. The oxidative imbalance that leads to ferroptosis can activate ISR in several ways. However, the relationship between ISR and ferroptosis remains underexplored.

Multiple lines of evidence suggest crosstalk between ISR and ferroptosis (Figure 6C). Deficiency of selenium sensitizes cells to ferroptosis [171,172]. tRNA specific to selenium participates in the synthesis of GPX4, a selenoprotein that regulates ferroptosis [200]. In selenium deficiency, accumulation of selenium-specific uncharged tRNA possibly activates the stress kinase GCN2 suggesting a crosstalk between stress kinase and ferroptosis. During metabolic or oxidative stress, a cytosolic Nicotinamide Adenine Dinucleotide Phosphate (NADPH) phosphatase called MESH1 (Metazoan SpoT Homologue 1) is induced, depleting NADPH and resulting in ferroptosis [173,174]. In addition, MESH1 depletion-induced ER stress leading to activation of PERK and subsequent phosphorylation of eIF2α [174] suggests a crosstalk between ISR and ferroptosis; however, the mechanism and significance of this potential crosstalk remain unexplored. Iron accumulation (as in hemochromatosis) also sensitizes cells to ferroptosis by enhancing lipid peroxidation [175,176,201]. In contrast, as discussed earlier, heme (a form of iron) sufficiency inhibits activation of the stress kinase HRI. This represents another potential mechanism for crosstalk between ISR and ferroptosis. Lower amounts of GSH sensitize cells to ferroptosis. However, an increased amount of GSH has been found in West Nile Virus (WNV) infected cells that protects cells from oxidative damage [202]. In addition, increased amounts of GSH are correlated with the absence of stress granules in WNV-infected cells [202]. These observations suggest a possible crosstalk between ISR and ferroptosis during viral infection as well. Taken together, several avenues for crosstalk between ISR and ferroptosis are present but remain unexplored. These avenues represent opportunities for exciting new discoveries in the future.

## 6. Conclusions

Various pathogens and cancer cells activate stress kinases leading to the formation of SGs as an attempt to maintain cellular homeostasis. In addition, stress granules sequester components of apoptosis (executioner caspases 3/7) [203], pyroptosis [9] and necroptosis [40,204] to favor pro-survival pathways. Sustained exposure to stress triggers one or the other forms of the RCD pathway. The crosstalk between different cell death types is emerging. Therefore, it is intriguing to know how stress kinases bridge with RCDs and how they can fine-tune different forms of cell death. Understanding the complex interaction between cellular stress and RCD will help to identify novel therapeutic targets.

## Data Availability

Not applicable.
